# Supplementary material for: Learning curve and surgical outcome of robotic assisted colorectal surgery with ERAS program
Source: Sci Rep. 2022 Nov 29;12:20566. doi: 10.1038/s41598-022-24665-w (PMC9709162; doi:10.1038/s41598-022-24665-w)
Supplement: Supplementary file 2 — Supplementary Information 2. [file 41598_2022_24665_MOESM2_ESM.zip › RAL Raw data-Ñ[▒K/054/OP_ñw╝╨░O▒Kñσ.pdf]

正式摘要(電子簽章：000000209230238)

W52 / W52

臺中榮民總醫院

Operation Note

[redacted]

- 手術日期：2021/01/20
- 手術醫師：VS 林俊余 , CR 林陳彥 , R 林辰祐
- Anesthesia : GE
- Position : Lithotomy
- Aseptic agents : Betadine Aqua , Zephnan

Diagnosis

- Pre-operative diagnosis : S-colon diverticulosis with recurrecnt diverticulitis events and huge liver cysts
- Post-operative diagnosis : s/p robotic anterior resection + fenestration for hepatic cyst

Surgical Indication

- For removing the infection focus

Operation

- 1. Robotic (XI) assisted anterior resection.
  # Fenestration for hepatic cyst was done under GS specialist.
  2. Intraoperative colonoscopy : (-)
  3. High ligation (-) : At IMA upper rectum branch. Left colic artery and IMV were preserved.
  4. Take down from the splenic flexure : (-)
  5. Robotic endoGIA (45-4.3) X2 + EEA (29mm) were applied to completed the end to end anastomosis.
  6. Indocyanine green agent was administered for testing during operation.
  7. No drainage tube was inserted.
  8. Hyalobarrier gel was applied below the wound over the LLQ of abdomen.

Finding

- 1. Multiple diverticulosis over S-colon.
  2. Adhesion was noted over the omentum and the peritoneum. Moderate adhesion was also presented between the S-colon and the nearby small bowel, which was compatible with her diverticulitis hx.
  3. No other gross tumor nor polyps was found
  4. No obvious ascites was noted
  5. Indocyanine green test result :
  A. Prior to the resection of rectum (Distal of the specimen) : About 27 seconds
  B. Prior to resection of proximal S-colon (Proximal of the specimen) : About 28 seconds
  C. After the anastomosis was nearly completed (Right before firing EEA) : About 23 seconds
  6. Bilateral doughnuts : intact ; Air leak test: Done and no air leak was found

Procedure

- 1. General endotracheal anesthesia was induced and then the patient was placed in modified Trendelenburg lithotomy position.
  2. Preoperative antibiotics were given. A Foley catheter and nasogastric tube were placed.
  3. The abdomen was prepped and draped in standard sterile fashion.
  4. GS specialist had performed their procedure to remove the hepatic cyst first.

- 5. We then taken over back for robotic AR after they had completed their operation.
  6. Laparoscopy was inserted from the supra umbilicus site (Already done by GS specialist, served as the 2nd arm port) and then another 8mm trocar was inserted over LUQ area of abdomen (Served as 1st arm port), also another 8mm one over RLQ area of abdomen (served as 4th arm port). A 12mm trocar was then inserted over RLQ (Served as the 3rd arm port). Finally, a 12mm trocar was inserted over RUQ area as an assist-port. The operative findings were mentioned as above.
  7. The sigmoid colon was held by grasper and the peritoneum overlying the mesentery was scored with electrocautery.
  8. The inferior mesentery artery was identified, ligated above the left colic artery with endoclips, and transected.
  9. Dissection was done above the Gerota's fascia and toward the peritoneal attachments.
  10. The colon was mobilized from retroperitoneum overlying the sacral promontory and into the pelvis.
  11. Peritoneal reflection was not resected.
  12. Distal descending colon and s-colon were freely mobilized after the mesoclon was all dissected.
  13. Indocyanine green agent was administered from IV line (See operative findings for more informations). The blood supply of the colon was examed carefully.
  14. The distal s-colon was divided with robotic endoGIA (45-4.3) X2.
  15. The mesocolon part of proximal s-colon was dissected as possible via the support of robotic instrument. Indocyanine green agent was administered from IV line again for testing.
  16. A new incision was made over LLQ area of abdomen. We approached into the peritoneal cavity by open method. S-colon was pulled out.
  17. The proximal colon was then taken out for carefully inspection. The remaining mesenteric fat was gently cleared from the terminal 2-3 mm of the bowel ends.
  18. The field was isolated with moist pad and the bowel opened and sized. It was determined that a 29 mm circular stapler could be accommodated. A purse string suture of 2-0 prolene was placed. The tumor-containing tissue was removed. Then the anvil was inserted, followed by the purse string suture. The colon was then put back to the abdominal cavity.
  19. Pneumoperitoneum was constructed again.
  20. The anal canal was irrigated with distil water and then the circular stapler was introduced through the anus and pierced through the upper area (The ventral site of the body) of the stapler line.
  21. The proximal bowel with anvil in place was engaged with the circular stapler. Indocyanine green agent was administered from IV line for the last time for testing.
  22. Then the stapler was then fired. Two complete donuts were obtained and the distal one was submitted to pathology for margin confirmation.
  23. The anastomosis was tested for leaks with irrigation solution and air introduced through the rectum and was found to be patent and intact.
  24. The fascia of all the trochar sites were closed with interrupted sutures via 1-O Vicryl.
  25. Hyalobarrier gel was applied below the wound over the LLQ of abdomen.
  26. The LLQ wound was closed with interrupted sutures via Num. 1 Vicryl. Massive N/S was applied to irrigation the wound.
  27. The wound was further closed with 3-O Vicryl, and the skin was applied with Medipore.
  28. The patient tolerated the procedure well and was brought to the POR in stable condition.
- Estimated blood loss: minimum
- 器械數量: 正確
- 紗布數量: 正確

Image

- PAINT
  - abdomen

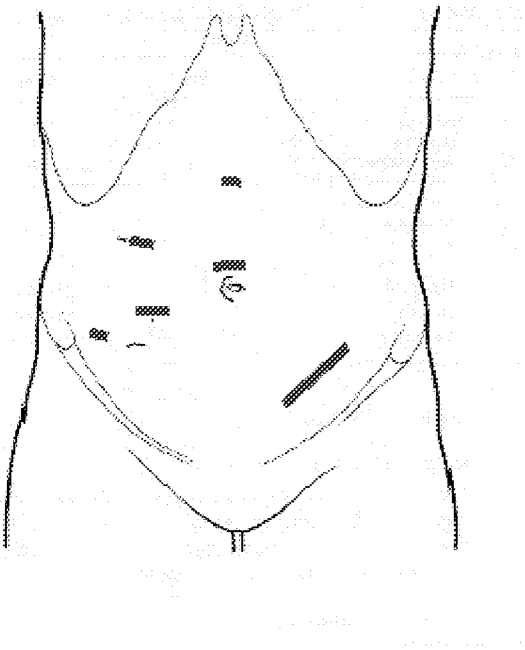

- colon & rectum

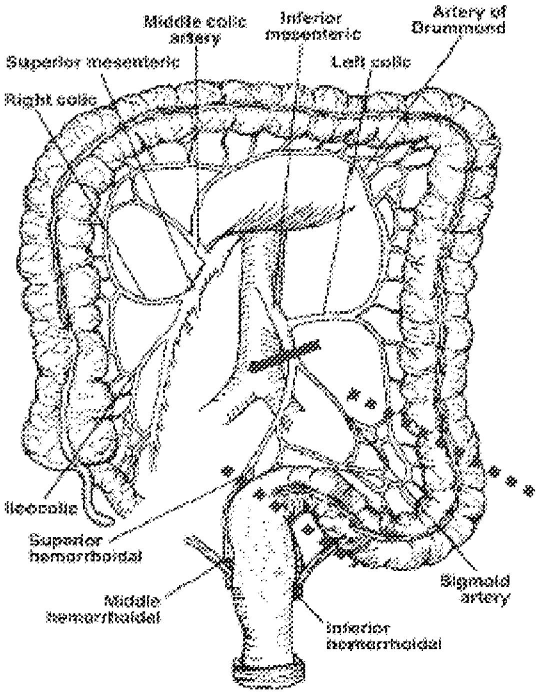

- The trochar wound as presented

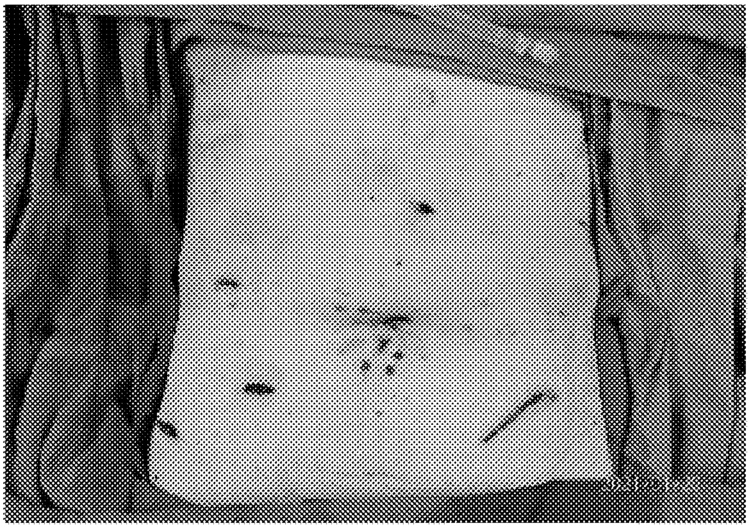

The resected S-colon

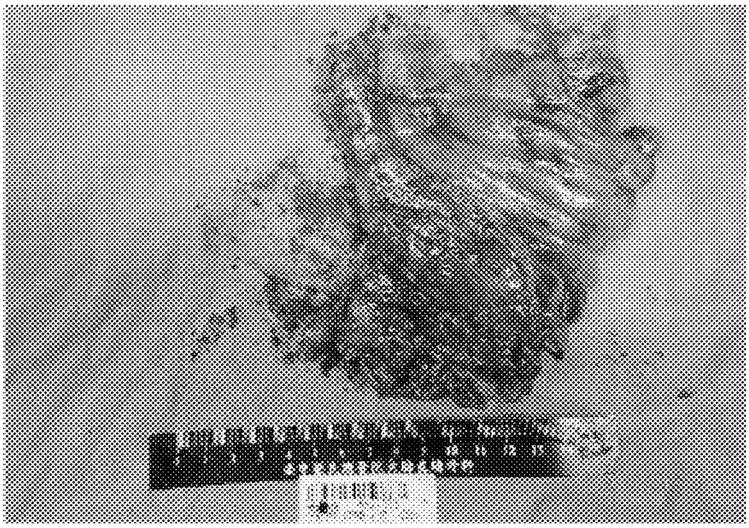

Diverticulosis could be clearly seen

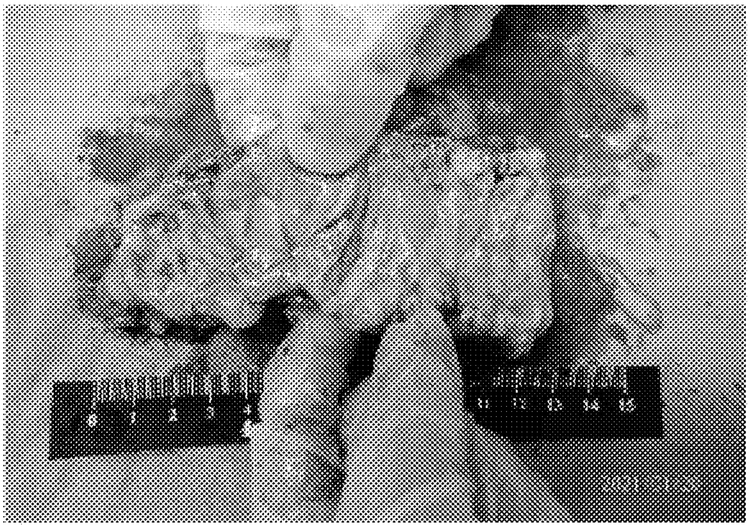

主治 2222H林俊余 / 醫師 2301H林陳彥

正式摘要(電子簽章：**000000209230238)**

W52 / W52

doctorRank:B
